# Hyper-physiologic mechanical cues, as an osteoarthritis disease-relevant environmental perturbation, cause a critical shift in set points of methylation at transcriptionally active CpG sites in neo-cartilage organoids

**DOI:** 10.1186/s13148-024-01676-0

**Published:** 2024-05-10

**Authors:** Niek G. C. Bloks, Amanda Dicks, Zainab Harissa, Rob G. H. H. Nelissen, Ghazaleh Hajmousa, Yolande F. M. Ramos, Rodrigo Coutinho de Almeida, Farshid Guilak, Ingrid Meulenbelt

**Affiliations:** 1 Dept. Biomedical Data Sciences, Section Molecular Epidemiology, Leiden University Medical Center, Einthovenweg 20, 2333 ZC Leiden, The Netherlands; 2 Washington University, Saint Louis, MO USA; 3 Shriners Hospitals for Children, Saint Louis, MO USA; 4 Department Orthopaedics, Leiden University Medical Center, Leiden, The Netherlands

**Keywords:** Osteoarthritis, DNA methylation, Chondrocytes, Mechanical loading, Environmental stressors

## Abstract

**Background:**

Osteoarthritis (OA) is a complex, age-related multifactorial degenerative disease of diarthrodial joints marked by impaired mobility, joint stiffness, pain, and a significant decrease in quality of life. Among other risk factors, such as genetics and age, hyper-physiological mechanical cues are known to play a critical role in the onset and progression of the disease (Guilak in Best Pract Res Clin Rheumatol 25:815–823, 2011). It has been shown that post-mitotic cells, such as articular chondrocytes, heavily rely on methylation at CpG sites to adapt to environmental cues and maintain phenotypic plasticity. However, these long-lasting adaptations may eventually have a negative impact on cellular performance. We hypothesize that hyper-physiologic mechanical loading leads to the accumulation of altered epigenetic markers in articular chondrocytes, resulting in a loss of the tightly regulated balance of gene expression that leads to a dysregulated state characteristic of the OA disease state.

**Results:**

We showed that hyper-physiological loading evokes consistent changes in CpGs associated with expression changes (ML-tCpGs) in *ITGA5, CAV1,* and *CD44,* among other genes, which together act in pathways such as anatomical structure morphogenesis (GO:0009653) and response to wound healing (GO:0042060). Moreover, by comparing the ML-tCpGs and their associated pathways to tCpGs in OA pathophysiology (OA-tCpGs), we observed a modest but particular interconnected overlap with notable genes such as *CD44* and *ITGA5.* These genes could indeed represent lasting detrimental changes to the phenotypic state of chondrocytes due to mechanical perturbations that occurred earlier in life. The latter is further suggested by the association between methylation levels of ML-tCpGs mapped to *CD44* and OA severity.

**Conclusion:**

Our findings confirm that hyper-physiological mechanical cues evoke changes to the methylome-wide landscape of chondrocytes, concomitant with detrimental changes in positional gene expression levels (ML-tCpGs). Since *CAV1, ITGA5,* and *CD44* are subject to such changes and are central and overlapping with OA-tCpGs of primary chondrocytes, we propose that accumulation of hyper-physiological mechanical cues can evoke long-lasting, detrimental changes in set points of gene expression that influence the phenotypic healthy state of chondrocytes. Future studies are necessary to confirm this hypothesis.

**Supplementary Information:**

The online version contains supplementary material available at 10.1186/s13148-024-01676-0.

## Introduction

Osteoarthritis (OA) is a complex, age-related multifactorial degenerative disease of the diarthrodial joints marked by impaired mobility, joint stiffness, pain, and a significant decrease in quality of life. Among other risk factors, such as genetics and age, hyper-physiological mechanical cues are known to play a critical role in the onset and progression of the disease [1]. OA is characterized by an imbalance in the articular chondrocytes’ anabolic and catabolic activities, impacting the integrity of the cartilage. Hyper-physiologic mechanical loading, as seen with post-traumatic injury, compresses articular chondrocytes and introduces catabolic signaling in chondrocytes [1, 2].

Previous studies have characterized the deregulated signaling pathways in articular chondrocytes in response to hyper-physiological mechanical cues with transcriptome-wide differential expression analyses. These studies show that hyper-physiologic mechanical cues significantly enhance cell apoptosis [3] and cellular senescence [4], increase catabolic gene expression [5], and reduce matrix production [6], whereas physiologic mechanical loading induces a broad anabolic response in the transcriptome that is associated with increased matrix formation [7]. Post-mitotic cells, such as articular chondrocytes, heavily rely on methylation at CpG sites to adapt to environmental cues and maintain phenotypic plasticity [8]. However, these long-lasting adaptations may eventually have a negative impact on cellular performance [9, 10]. We hypothesize that hyper-physiologic mechanical loading leads to the accumulation of altered epigenetic markers in articular chondrocytes resulting in a loss of the tightly regulated balance of gene expression to a dysregulated state characteristic of the OA disease state.

Here, we aimed to study the effect of hyper-physiological mechanical stress on changes in DNA methylation-driven set points of epigenetically regulated gene expression that potentially contribute to OA-related loss of the chondrocytes’ epigenetically controlled healthy maturational arrested phenotypic state. To this end, we employed an established human-induced pluripotent stem cell (hiPSC)-derived cartilage organoid model and studied the methylome and transcriptome-wide changes in response to previously assessed hyper-physiological mechanical loading conditions [11]. Using these techniques, we show that changes in the epigenetic set point of transcription in chondrocytes responding to hyper-physiological loading overlap with OA pathophysiology, further underlining their mutual role in evoking aberrant chondrocyte cellular functions.

## Results

### Characterization of experimental set-up to test the epigenome- and transcriptome-wide effects of hyper-physiological loading

To test the effects of hyper-physiological loading on the epigenetically regulated transcriptome, we employed a human-induced pluripotent stem cell (hiPSC)-derived neo-cartilage organoid model. hiPSCs were differentiated into chondrocytes using a previously established chondrogenic differentiation protocol [12]. To study the response of chondrocytes to hyper-physiological mechanical loading conditions, we have applied two different models that are commonly used in osteoarthritis research: [1] chondrocyte-derived (spherical) neo-cartilage constructs and [2] chondrocytes embedded in (cylindrical) agarose constructs. To distill the most consistent effects, we employed both models and performed a meta-analysis on the methylome-wide changes in response to hyper-physiological mechanical loading. Successful differentiation toward chondrocytes and the production of neo-cartilage were confirmed by protein immunolabeling of collagen II (COLII) and collagen VI (COLVI), as well as staining for sulfated glycosaminoglycans (sGAGs) (Additional file 1: Fig. S1A).

To test the hypothesis that injurious mechanical stress can alter the epigenetically regulated transcriptome, both of the organoid models were exposed to hyper-physiologic mechanical loading (total n = 26) alongside unloaded controls (total n = 27) [11]. Methylome- and transcriptome-wide profiles resulting from the jointly analyzed neo-cartilage models, together with real-time quantitative polymerase chain reaction (RT-qPCR), immunohistochemistry (IHC), and dimethyl methylene blue (DMMB) assays, resulting from the spherical neo-cartilage organoids were measured 12 h after mechanical stimulation. First, we characterized the response of neo-cartilage organoids to hyper-physiological loading conditions by targeted analysis using RT-qPCR of catabolic and anabolic cartilage markers and mechano-sensors (Table 1). Similar to other mechanically induced injurious in vitro and in vivo models of OA [4, 13], expression of anabolic *ADAMTS5* significantly increased in response to hyper-physiological loading. This finding suggests that hyper-physiological loading conditions induced a catabolic response in neo-cartilage organoids. Additionally, there was an increase in *PIEZO1* expression, which is hypothesized one of the main transducers of hyper-physiological mechanical loading [14]. Nonetheless, the staining intensity of COLII and COLVI, as well as sGAG deposition normalized to DNA content, showed no significant change in response to hyper-physiological loading conditions (Additional file 1: Fig. S1B-C).

**Table 1:** Effects of mechanical loading on general OA markers

Gene	Effect of Mechanical Loading	
	beta	*p*-value
*Catabolic*		
*MMP13*	0.41	0.94
*MMP3*	1.71	0.08
*ADAMTS5*	0.14	** < 0.001**
*Anabolic*		
*COL2A1*	0.03	0.87
*ACAN*	0.17	0.72
*Hypertrophic*		
*COL10A1*	-0.2	0.18
*Mechanosensory ion channels*		
*PIEZO1*	0.33	**0.03**
*PIEZO2*	0.09	0.61
*TRPV4*	0.02	0.91

### Methylome-wide response to hyper-physiological mechanical loading conditions

**Fig. 1:**
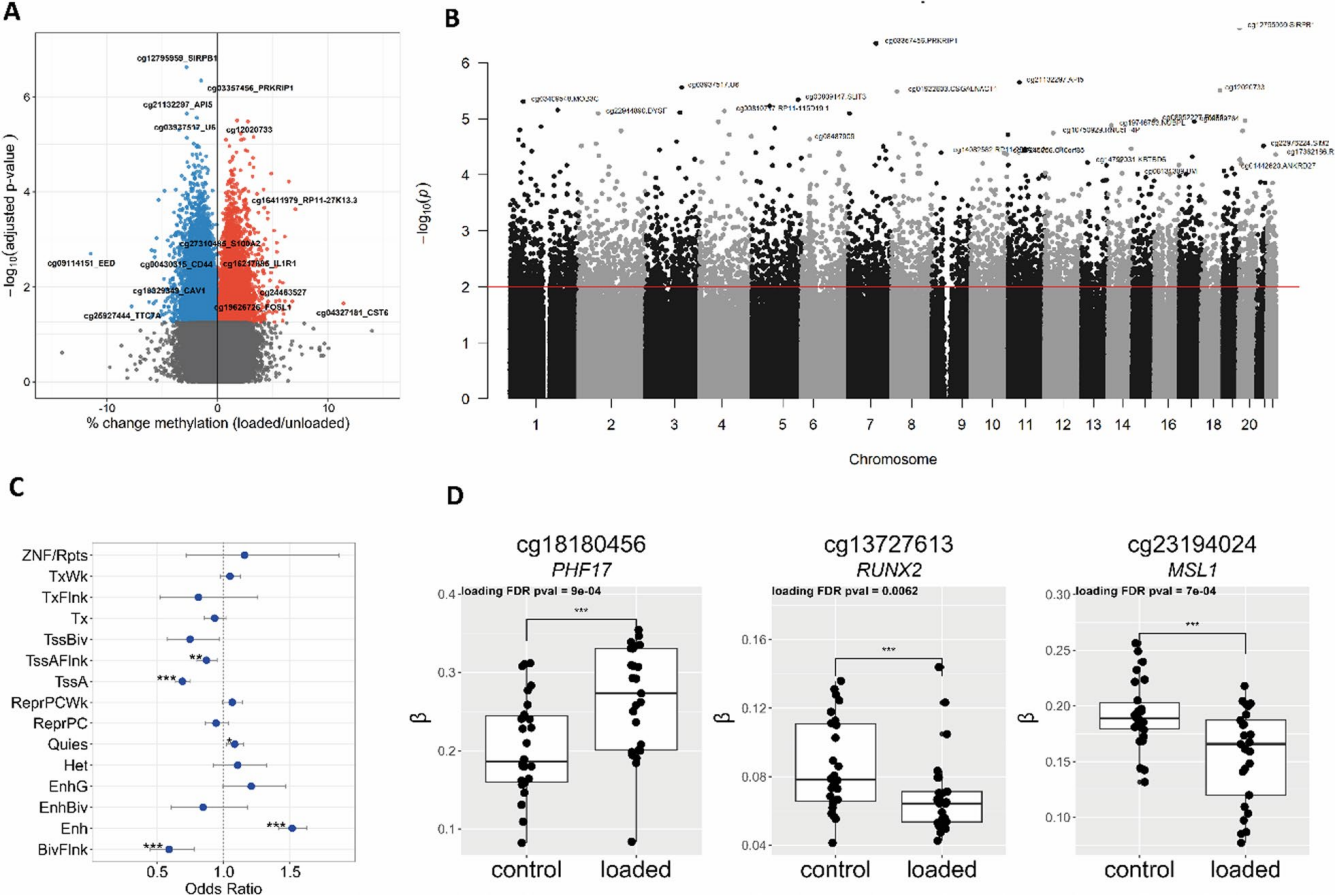
Effect of hyper-physiological loading on the genome-wide methylation **A** A volcano plot of the methylome-wide response to hyper-physiological loading conditions. Red dots denote CpG sites mapped to a gene body with increased methylation, FDR < 0.01, and blue dots represent CpG sites mapped to a gene body that are de-methylated in response to hyper-physiological loading conditions as determined by MEAL. **B** Manhattan plot of differentially methylated CpG sites with their genomic mapped genes. The horizontal red line represents the FDR < 0.05 threshold. **C** Enrichment of significant DMs within chromatin states; active transcription start site (TSS), proximal promoter states (TssA, TssAFlnk), a transcribed state at the 5′ and 3′ ends of genes showing both promoter and enhancer signatures (TxFlnk), actively transcribed states (Tx, TxWk), enhancer states (Enh, EnhG), and a state associated with zinc finger protein genes (ZNF/Rpts). The inactive states consist of constitutive heterochromatin (Het), bivalent regulatory states (TssBiv, BivFlnk, EnhBiv), repressed Polycomb states (ReprPC, ReprPCWk), and a quiescent state (Quies) **D** Notable example of differentially methylated mapped CpGs in response to hyper-physiological mechanical loading conditions. The box plots represent the 25th, 50th, and 75th percentiles, and whiskers extend to 1.5 times the interquartile range. Individual samples are depicted by black dots in each graph. *FDR < 0.05, **FDR < 0.01, ***FDR < 0.001

Following Illumina EPIC array analyses and quality control (QC), we obtained robust methylation data of 807,655 CpGs for the two models to determine differential DNA methylation in response to hyper-physiological loading by meta-analysis. In total, we detected 6830 differentially methylated (DM) CpG sites **(**FDR < 0.01**, **Fig. 1A; Additional file 2: Table S1) and plotted them across the genome annotated with the GENCODE basic V12 database **(**Fig. 1B**)** [15]. Notable examples of highly significant DM CpG sites are cg27310485 annotated to calcium binding protein *S100A2* (beta = 0.01, FDR = 7.8X10^−4^), cg16217885 annotated to inflammatory receptor *IL1R1* (beta = 0.01, FDR = 1.2 × 10^–3^), and cg12795959 annotated to *SIRPB1* (beta = -0.03, FDR = 2.4 × 10^–7^). As shown in Fig. 1B, we recognized some skyscrapers suggesting differentially methylated regions (DMRs). Upon defining DMRs as 3 or more DM CpGs with an inter-CpG distance of < 1 kb and allowing for 3 non-DM CpGs in the complete DMR [16], these DMRs did not reach statistical significance. To gain insight into the functional aspects of DM CpGs, their enrichment to chromatin states was analyzed [17]. As shown in Fig. 1C**,** DM CpG sites with hyper-physiological loading were significantly enriched within chromatin regions associated with active transcription start site (TSS) proximal promoter states TssAFlnk (OR = 0.87, FDR = 1.8 × 10^−3^)*,* transcription start sites (TssA) (OR = 0.69, FDR = 4.7 × 10^−21^), enhancer states (Enh) (OR = 1.51, FDR = 6.5 × 10^−31^), bivalent regulatory states (BivFlnk) (OR = 0.59, FDR = 2.0 × 10^−4^), and a quiescent state (Quies) (OR = 1.08, FDR = 6.3 × 10^−3^). The highly significant enrichment for DM CpGs in Enh suggests that mechanical loading-induced differential methylation, particularly at CpG sites that reside in genomic regions involved in the regulation of gene expression. Notable examples of highly significant DM CpG sites that mapped to such regulatory regions of gene expression were cg18180456 annotated *PHF17* encoding Jade Family PHD Finger 1 involved in histone acetylation (beta = − 0.0632, FDR = 8.8 × 10^−5^), cg13727613 annotated *RUNX2* encoding a well-known transcription factor detrimental to cartilage, (beta = -0.021, FDR = 6.2 × 10^−4^), and cg23194024 annotated *MSL1* encoding a component of the histone acetyltransferase complex responsible for the majority of histone H4 acetylation (beta = -0.032, FDR = 6.9 × 10^−5^) **(**Fig. 1D**)**.

### Transcriptionally active CpG sites

**Fig. 2:**
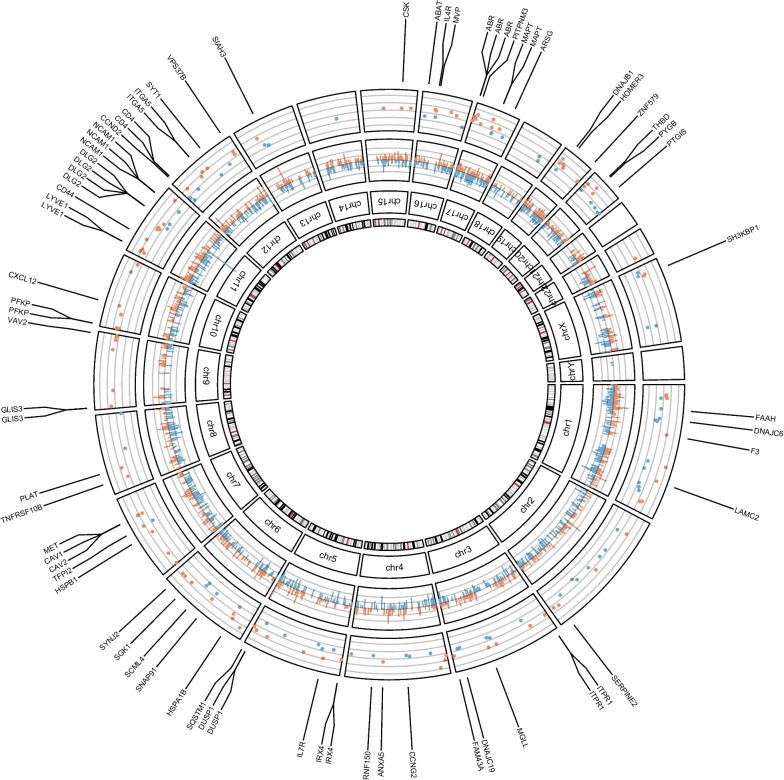
Circos plot of transcriptionally active DM CpG sites responding to hyper-physiologic mechanical loading showing the genomic distribution of the DM CpG sites and positional DE genes. The inner circle displays the chromosomes. The middle circle displays the change in percentage of the methylation of the 2492 DM CpGs that mapped to a gene site. Blue bars depict de-methylated CpG sites, and red bars depict increased methylated CpG sites. (FDR < 0.01) The outside circle displays the ^2^log fold change of the 169 unique DE ML-tCpG-Genes. Blue dots depict downregulation, and red dots depict upregulation. (FDR < 0.05)

**Fig. 3:**
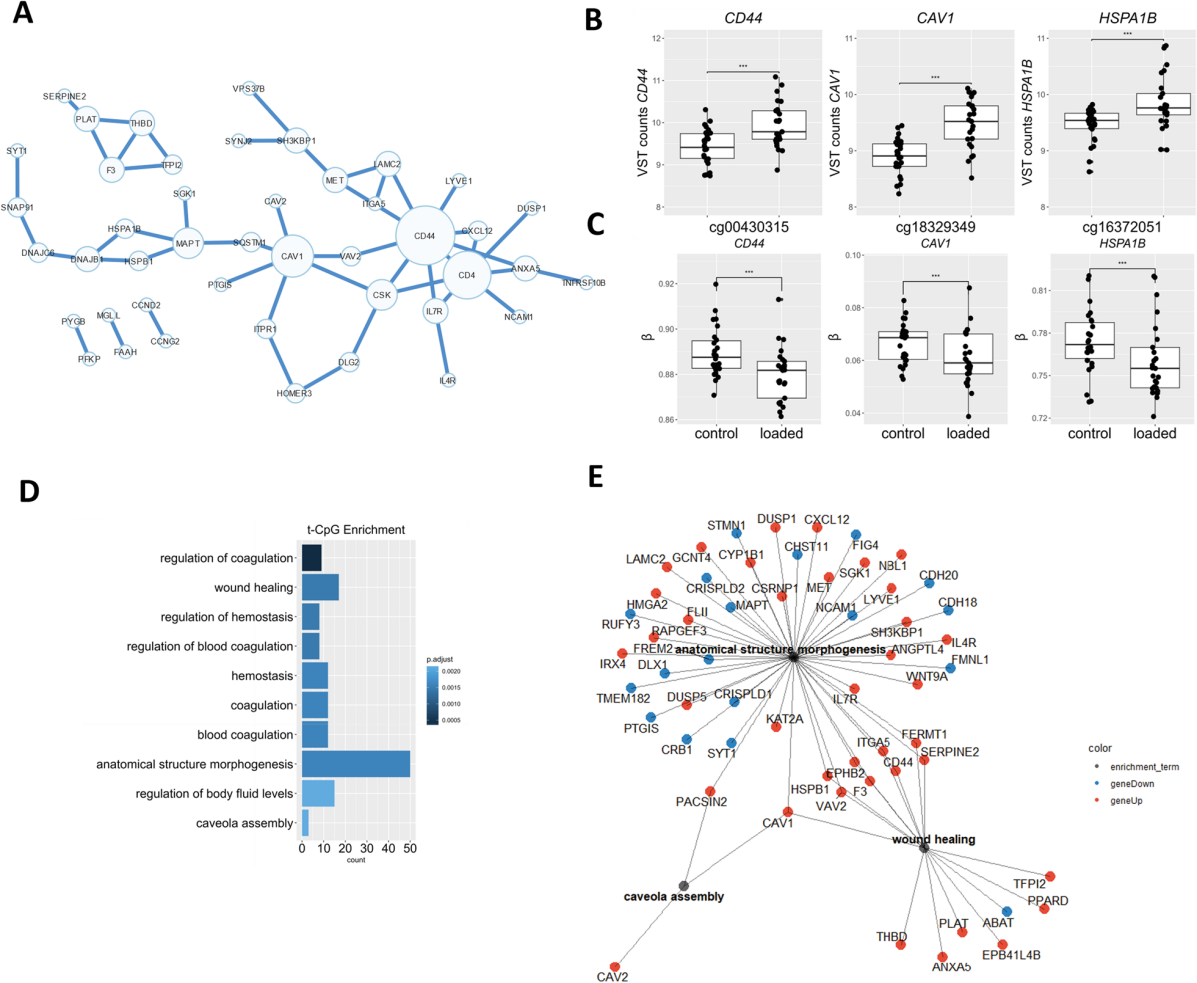
Epigenetically regulated transcriptome pathway enrichment analysis. **A** Protein–protein network of ML-tCpG—genes as determined by STRING-DB. **B** Differential gene expression of genes to which a DM CpG was mapped. **C** Differential methylation of CpG sites mapped to the gene body. The box plots represent 25th, 50th, and 75th percentiles, and whiskers extend to 1.5 times the interquartile range. Individual samples are depicted by black dots in each graph. *FDR < 0.05, ***FDR < 0.001. **D** Top 10 most significantly enriched pathways of epigenetically regulated DEG. FDR < 0.05. **E** Gene-pathway network of notable enriched pathways where lines depict the relationship between the genes and the pathways determined by enrichment analysis. Blue dots depict downregulated DEGs in response to hyper-physiologic mechanical loading conditions, and red dots depict upregulated DEGs in response to hyper-physiologic mechanical loading conditions

To biologically interpret the DM CpG sites in response to hyper-physiological loading more specifically, we next integrated a previously assessed RNA sequencing dataset [18] of the same experiment. To prioritize DM CpG that likely affect gene expression, we first prioritized, among the DM CpG sites, those that mapped to genes within 200 or 1500 bp of the transcription start site (TSS200, TSS1500), located within the 3′ or 5′ UTR regions, or CpG sites that were exon bound. This resulted in 2492 CpG-gene pairs (Fig. 2; Additional file 2: Table S2). As shown in Fig. 2, around multiple genes such as *ITGA5*, *DLG2,* and *ABR* multiple DM CpGs are clustered As is evident by the chromosomal clustering of multiple DM CpG sites at positioning of Next, among the 2492 DM CpG sites, we prioritized those that mapped to a gene that was also differentially expressed in response to hyper-physiological loading conditions based on FDR correction for the number of genes overlapping with mapped CpGs. This selection of the most likely transcriptionally active DM CpGs, henceforth defined as mechanical loading-induced, transcriptionally active CpGs (ML-tCpGs), consisted of a total of 208 ML-tCpGs that mapped at TSS200 (15.4%), TSS1500 (28.5%), 3′UTR (13.65%), 5′UTR (32.2%), and 1st Exon (5.78%) or were Exonbound (4.3%) (Additional file 2: Table S3). As shown in Fig. 2 and Additional file 2: Table S3, these 208 ML-tCpGs were connected to 169 unique differentially expressed (DE) genes. As shown in Fig. 3A, 57 of the 169 genes showed a strong protein–protein interaction (FDR = 2.4 × 10^–5^) as determined by STRING-DB. Examples of highly connected ML-tCpGs-genes within this network are *HSPA1B, CD44,* and *CAV1* (Fig. 3B–C). To gain insight into the biological processes that are affected by the ML-tCpGs responding to hyper-physiological loading conditions, we performed pathway enrichment analysis of gene ontology biological processes (GO BP), KEGG, and Reactome (Additional file 2: Table S4) on all 169 unique genes that were associated with a ML-tCpG. Pathways such as anatomical structure morphogenesis (e.g., *ANGPTL4, WNT9A, HMGA2)*, wound healing (e.g., *HSPB1*, *SERPINE2, FERMT1)*, and caveola assembly (e.g., *CAV1*, *CAV2, PACSIN2)* were enriched.

### Overlapping key nodes in the network of tCpGs affected by mechanical loading and OA pathophysiology

To explore the role of the identified ML-tCpG-gene pairs in OA pathophysiology, we examined the overlap of our ML-tCpG-gene pairs with those previously reported tCpG-gene pairs associated with OA pathophysiology, i.e., differentially expressed between lesioned and preserved cartilage from OA patients who underwent a joint replacement surgery [19]. Although, the overlap was modest (n = 8 of 142 OA-tCpG-gene pairs) (Additional file 1: Fig. S3), the ML-tCpG genes that did overlap with OA-tCpGs, *CD44*, *ITGA5,* and *CAV1,* are central and particularly interconnected genes in the network of both ML-tCpG-gene pairs and OA-tCpG-gene pairs (Fig. 3A; Additional file 1: Fig. S2). Taken together, we showed that hyper-physiological loading resulted in changes in tCpG-gene pairs that are both located within gene regulatory chromatin states and that are central and responsive to changes that occur during OA pathophysiology.

### Correlation of tCpGs with clinical OA phenotypes

Finally, we set out to gain insight into the clinical relevance of altered epigenetic control of the identified ML-tCpG. Therefore, we determined the association of ML-tCpGs overlapping with OA-related epigenetically regulated genes [8] to phenotypic traits in the respective preoperative radiograph of the OA patient (the RAAK study). This subset of ML-tCpGs regulated *PFKP, CD44, CAV1, SVIL,* and *CY1BP1.* Next, to assess how methylation levels of these genes relate to OA severity, methylation levels in preserved cartilage from joint replacement surgeries of these overlapping ML-tCpGs were correlated with OA severity as determined by the Kellgren–Lawrence grading scale (KL-score), adjusted for BMI and age. We found that both *CD44* (beta = -0.053, P = 0.048) and *PFKP* (beta = -0.111, P = 3.12X10^−3^) methylation levels were negatively associated with the KL-score, suggesting indeed that mechanical loading-induced alterations in epigenetic set points of expression are associated to the OA disease state of the patient.

## Discussion

The goal of this study was to determine the effect of hyper-physiological mechanical loading on changes in stable set points of epigenetically regulated gene expression (ML-tCpGs) that could contribute to the long-lasting, detrimental changes in chondrocytes that are characteristic of an OA phenotype. To this end, we employed two human-induced pluripotent stem cell (hiPSC)-derived neo-cartilage organoid models for robust readouts and studied the methylome- and transcriptome-wide changes in response to hyper-physiological mechanical loading conditions. We showed that hyper-physiological loading evokes consistent changes in ML-tCpGs associated with expression changes in *ITGA5, CAV1,* and *CD44,* among other genes, which together act in pathways such as anatomical structure morphogenesis (GO:0009653) and response to wound healing (GO:0042060). Moreover, by comparing the ML-tCpGs and their associated pathways to tCpGs in OA pathophysiology, we observed a modest but particular interconnected overlap with notable genes such as *CD44* and *ITGA5.* These genes could indeed represent lasting detrimental changes to the phenotypic state of chondrocytes due to mechanical perturbations that occurred earlier in life. The latter is further suggested by the association between methylation levels of ML-tCpGs mapped to *CD44* and OA severity.

Since injurious mechanical loading is considered an important driver of the onset and progression of the OA, here, we studied for the first time whether injurious mechanical loading evokes stable, detrimental changes to chondrocyte phenotypic states. In doing so, we revealed that DM CpGs are particularly enriched in transcription start sites and enhancers suggesting that the DM CpG sites evoke changes in gene transcription. However, we cannot exclude the epigenetic regulatory effects of *in trans* tCPGs. To allow biological interpretation of the DM CpGs in response to hyper-physiological loading, we integrated RNA sequencing data from the same experiment. We showed that significant differential CpG-gene pairs with hyper-physiological loading occurred, particularly near genes OA-relevant genes (e.g., *ITGA5, CD44, CAV1, WNT9A,* and *HMGA2)*. The relevance of mechanically induced expression of *ITGA5* is that the binding of matrix fragments such as fibronectin to integrin α5β1 heterodimer activates a pro-catabolic response [20]. Also, *CD44* plays a role in matrix catabolism by degrading hyaluronic acid in articular cartilage [21], while catabolic stress has been shown to upregulate *CAV1,* coding for caveolin-1, which has been linked to chondrocyte senescence [22]. Genes like *WNT9A* as well as *HMGA2* are reported OA risk genes [23]. Nonetheless, the design of our study does not justify a direct causal relationship between DM at ML-CpGs and differential gene expression. To further confirm a direct causal relationship between CpG-specific methylation levels and gene expression, a CRISPR-Cas9-DNMT/TET1 guided manipulation of methylation levels of the identified CpG sites mapped to *CD44, PFKP*, *SVIL,* and *CY1BP1*, is warranted.

Here we have combined genome-wide methylation and RNA-seq analysis of hiPSC-derived chondrocytes either in deposited (spherical) neo-cartilage or embedded in (cylindrical) agarose, which currently are the most commonly used models in osteoarthritis research [7, 11, 14, 24]. The advantage of the spherical neo-cartilage model is that it contains an extracellular matrix deposited by the chondrocytes. Herein the response of the chondrocyte to the mechanical perturbation likely reflects changes in the chondrocyte–matrix interaction. Additionally, it allows for evaluating responses of mechanical loading on sGAGs and other matrix constituents by histology. Henceforth, this model has been used to evaluate the effects of hyper-physiologic mechanical loading conditions on matrix properties. On the other hand, the strain distribution on the spherical pellets, hence chondrocytes, is less equal and could have reduced power or introduce bias. The advantage of the cylindrical-shaped model, for that matter, allows for an equal strain distribution, hence a more precise relation between the applied stress and the response of the chondrocytes to the deformation. By performing a meta-analysis on the methylome-wide landscape of these two models, we aimed to distill the most consistent and robust effects in the molecular response to hyper-physiologic mechanical loading conditions.

Founded by the notion that early environmental challenges could evoke long-lasting changes to methylation at tCpG sites, resulting in detrimental changes to set points of gene expression, we sought evidence that ML-tCpGs are associated with previously assessed, OA-related differences in methylation in articular cartilage (OA-tCpGs) [8]. We showed that genes such as *CAV1, CD44,* and *ITGA5* appeared to be identically changed, highly interconnected, and central to both the OA-tCpGs and ML-tCpGs networks. As such, it is tempting to suggest that mechanical injurious loading can indeed contribute to stable and detrimental changes to the phenotypic state of chondrocytes eventually making the chondrocyte prone to an OA phenotype. Nonetheless, this hypothesis needs to be further investigated by confirming that *CD44* upregulation leads to detrimental downstream effects of chondrocytes toward an OA chondrocyte phenotype.

After subjecting the neo-cartilage organoids to a single episode of mechanical injurious loading, the epigenetic and transcriptomic profiles were captured 12 h later. This loading regime was based on a previous study, which optimized the loading regime to induce catabolic signaling in neo-cartilage organoids. [11] Although this loading regime resulted in many changes in methylation and associated expression, we did not observe changes in matrix content. The fact that histological changes were not visible is likely due to the relatively early time point and/or the intrinsic insensitivity of identifying changes in protein levels. Moreover, although changes in methylation at CpG sites are generally considered stable and long-lasting [10], the fact that we only measured methylation at 12 h post-stimulus did not allow us to confirm the duration of the change in methylation, and future studies may wish to address this question directly. Additionally, this model of hyper-physiological loading in hiPSC neo-cartilage organoids can be expanded to define and apply more beneficial mechanical loading regimes, further unraveling the shift in molecular mechanisms underlying the normal physiological response to loading, and potentially counteract the detrimental changes in set points in gene expression as induced by more damaging loading regimes. Such insights might further aid in the development of treatment strategies.

## Conclusion

Together, the current study confirms that hyper-physiological mechanical cues evoke changes to the methylome-wide landscape of chondrocytes, concomitant with detrimental changes in positional gene expression levels (ML-tCpGs). Since *CAV1, ITGA5,* and *CD44* are subject to such changes and are central and overlapping with OA-tCPGs of autologous cartilage, we advocate that accumulation of hyper-physiological mechanical cues can evoke long-lasting, detrimental changes in set points of gene expression that eventually affect the phenotypic healthy state of chondrocytes. Future studies are necessary to confirm this hypothesis.

## Methods

### Experimental design

The objective of the current study was to study the effects of hyper-physiologic mechanical loading conditions on the epigenetically regulated transcriptome. Here, we employed an hiPSC-derived neo-cartilage organoid model that was exposed to hyper-physiological mechanical loading conditions. These samples were then analyzed using 850 k EPIC array and RNA sequencing.

### hiPSC line and cell culture

An hiPSC line as described earlier was used [12]. In short, the RVR-hIPSC line was retrovirally reprogrammed from human foreskin BJ fibroblasts and characterized. The hiPSCs were maintained under standard conditions (37 °C, 5% CO_2_) on Matrigel (Corning, cat # 356237, New York, US) coated plates and refreshed daily with TeSR-E8 medium (STEMCELL Technologies, Vancouver, Canada) with 0.5% penicillin–streptomycin (P/S; Gibco Landsmeer, the Netherlands) upon reaching approximately 70% confluence.

### hiPSC differentiation to induced chondrocytes

Generation of hiPSC-derived chondrocytes was based on a protocol previously described [12], which was shown to result in the formation of tissue similar to young human cartilage [25–27]. When hiPSCs reached 60% confluence, the culture medium was switched to mesodermal differentiation (MD) medium, composed of IMDM GlutaMAX (IMDM; Thermo Fisher Scientific, St Louis, MO) and Ham’s F12 Nutrient Mix (F12; Sigma-Aldrich Zwijndrecht, the Netherlands) with 1% chemically defined lipid concentrate (Gibco Landsmeer, the Netherlands), 1% insulin/human transferrin/selenous (ITS + ; Corning), 0.5% penicillin–streptomycin (P/S; Gibco Landsmeer, the Netherlands), and 450 μM 1-thioglycerol (Sigma-Aldrich, Zwijndrecht, the Netherlands). Before induction of anterior primitive streak (day 0), hiPSCs were washed with wash medium (IMDM/F12 and 0.5% P/S) and then fed with MD medium supplemented with activin A (30 ng/ml; Stemgent), 4 μM CHIR99021 (CHIR; Stemgent, Zwijndrecht, the Netherlands), and human fibroblast growth factor (20 ng/ml; FGF-2; R&D Systems) for 24 h. Subsequently, the cells were washed again with wash medium, and paraxial mesoderm was induced on day 1, by MD medium supplemented with 2 μM SB-505124 (Tocris, Bristol, UK, 3 μM CHIR, FGF-2 (20 ng/ml), and 4 μM dorsomorphin (Tocris, Bristol, UK, for 24 h. Before induction of early somite (day 2), cells were washed with wash medium, and then cells were fed with MD medium supplemented with 2 μM SB-505124, 4 μM dorsomorphin, 1 μM C59 (Cellagen Technology), and 500 nM PD173074 (Tocris, Bristol, UK), for 24 h. Subsequently, cells were washed with wash medium, and for induction of sclerotome, cells (days 3 to 5) were fed daily with MD medium supplemented with 2 μM purmorphamine (Stemgent Zwijndrecht, the Netherlands) and 1 μM C59. To induce chondroprogenitor cells (days 6 to 14), cells were washed briefly with wash medium and fed daily with MD medium supplemented with human bone morphogenetic protein 4 (BMP-4; 20 ng/ml; Miltenyi Biotec, Leiden, the Netherlands).

Monolayer cultured hiPSC aggregates present at day 14 of the differentiation were washed with MD medium, dissociated with Gentle Cell dissociation medium (Stem Cell, Vancouver, Canada) and centrifuged for 5 min at 300G. Cell aggregates were subsequently maintained in chondrogenic differentiation (CD) medium containing Dulbecco’s modified Eagle’s medium/F12 (Gibco Landsmeer, the Netherlands, supplemented with 1% ITS + , 55 μM 2-mercaptoethanol (Gibco Landsmeer, the Netherlands), 1% non-essential amino acids (Gibco Landsmeer, the Netherlands), 0.5% P/S, L-ascorbate-2-phosphate (50 μg/ml; Sigma-Aldrich, Zwijndrecht, the Netherlands), L-proline (40 μg/ml; Sigma-Aldrich, Zwijndrecht, the Netherlands), ML329 (1 µM; CSNpharm), C59 (1 µM; Tocris, Bristol, UK),, and transforming growth factor–β3 (10 ng/ml; PeproTech, London, UK) for 30 days while refreshing medium every 3 to 4 days.

### Neo-cartilage organoid models

Two different chondrogenic constructs were used for downstream analysis; either these chondrogenic constructs were directly used for further experiments or they were dissociated using collagenase II, encapsulated in 2% w/v agarose at 30 million cells/ml, and cultured for 14 days with CD creating cylindrical-shaped constructs. Across the independent differentiations a total of 53 samples were used for molecular profiling via RNA-seq and methylation analysis divided loaded samples (n = 26) alongside unloaded controls (n = 27). For validation experiments using RT-qPCR analysis only, the spherical model was employed. Both models were used for methylome-wide analysis as described below.

### Mechanical loading

The spherical-shaped neo-cartilage constructs were mechanically loaded using a MACH-1 mechanical testing device (Biomomentum, Laval, Canada), at a rate of 5 Hz with 20% sinusoidal peak-to-peak strain for 10 min as described earlier [11]. The cylindrical constructs were loaded with a custom-build mechanical loading device, with the same loading regime. After mechanical loading, we have placed the neo-cartilage organoids back into CD medium excluding transforming growth factor–β3 to prevent interference of its anabolic response with the response to mechanical loading.

### sGAG measurement

Sulfated glycosaminoglycan (sGAG) concentrations in the neo-cartilage organoids (µg sGAG/µg DNA) were measured using the Farndale Dimethyl Methylene Blue (DMMB, Sigma, Zwijndrecht, the Netherlands) method [28]. Chondroitin sulfate (Sigma, Zwijndrecht, the Netherlands) was used as a reference standard. Absorbance was measured at 535 and 595 using a microplate reader (Synergy HT, Biotek, Winooski, VT, USA). Neo-cartilage sGAG concentrations were corrected for DNA content measured with the Qubit® 2.0 Fluorometer (Invitrogen™, Carlsbad, CA, USA) using the dsDNA HS Assay Kit (Invitrogen™, Carlsbad, CA, USA).

### Histology and immunohistochemistry

Neo-cartilage samples were fixed in 4% formaldehyde and embedded in paraffin. Sections were stained with Alcian Blue (Sigma-Aldrich, Zwijndrecht, the Netherlands) and Nuclear Fast Red (Sigma-Aldrich, Zwijndrecht, the Netherlands). Deposition of collagen VI and collagen II in the neo-cartilage constructs was visualized immunohistochemically using a polyclonal antibody for COL6A1 (abcam ab6588), a primary sub-unit of COLVI, and a polyclonal antibody for COL2A1 (abcam ab34712), a primary sub-unit of COLII., antigen retrieval was done by treating deparaffinized sections with proteinase K (5 µg/ml, Qiagen Venlo, the Netherlands) and hyaluronidase (5 mg/ml, Sigma Zwijndrecht, the Netherlands). Sections were incubated overnight with the primary antibodies, followed by incubation with a HRP conjugated secondary antibody (ImmunoLogic). Peroxidase binding for collagen VI was visualized using diaminobenzidine, and sections were counterstained with hematoxylin.

### RT-qPCR

Per sample, two replicate neo-cartilage pellets were collected in TRIzol (Invitrogen™, Carlsbad, CA, USA), and RNA was isolated using the RNeasy Mini Kit (Qiagen, Venlo, the Netherlands) according to the manufacturer’s protocol. DNA contamination was removed by treating the RNA with Rnase-Free DNase. RNA quality (A260/280: 1.7–2.0) was assessed using a nanodrop. RNA concentrations were measured with the Qubit® 2.0 Fluorometer (Invitrogen™, Carlsbad, CA, USA) using the RNA HS Assay Kit (Invitrogen™, Carlsbad, CA, USA), with an A260/280 between 1.7–2.0. RNA was reverse transcribed into cDNA using the Transcriptor First Strand cDNA Synthesis Kit (Roche, Basel, Switzerland). cDNA was amplified using FastStart SYBR Green Master (Roche, Basel, Switzerland), and mRNA expression was measured in triplicates in a MicroAmp™ Optical 384-Well Reaction Plate (ThermoFisher Scientific, Landsmeer, the Netherlands), using the QuantStudio™ Flex Real-Time PCR system (Applied Biosystems™, Foster City, CA, USA), with the following cycling conditions: 10 min 95 °C; 10 s 95 °C, 30 s 60 °C, 20 s 72 °C (45 cycles); 1 min 65 °C and 15 s 95 °C. Primer efficiency was tested using a cDNA dilution series, and primers were considered efficient with an efficiency between 90 and 110%. − ΔCt expression levels were calculated using two housekeeping genes *GAPDH* and *SDHA* with the following formula: *ΔCt* = *Ct (gene of interest)—Ct (average housekeeping genes)*. Both housekeeping genes were stably expressed in this model. Fold changes were calculated using the 2^−ΔΔCt^ method with *ΔΔCt* = *ΔCt (MS) – ΔCt (Control)*.

### Methylation data analysis

DNA methylation was assessed using the Illumina Infinium Methylation EPIC (850 K) BeadChip according to GenomeScan’s standard operating procedures (SOPs) based on the Illumina Infinium II Protocol. To analyze methylation array data (MethylationEPIC 850 k array), the MethylAid R script [29] with the default settings was used for data quality assessment. All samples showed a detected CpG above 95%. The minfi.v_1.36.0 R package [30] was used to pre-process the data. We removed any probe that have failed in one or more samples (*p* < 0.01). Probe level intensities were quantile normalized across samples prior to calculation of the *ß*-values. MethylToSNP was used to filter SNPs. This method looked for patterns in methylation array data and identified methylation probes with SNP-like patterns. The method removes outliers, which adds robustness to the analysis and is enabled by default. A confidence score was calculated to show how close the observed pattern of methylation beta values was to a canonical case of a SNP in a homozygously methylated CpG locus. Additionally, MethylToSNP can overlap the SNPs identified in methylation data with known SNPs from dbSNP. The probes that have shown to be cross-reactive (demonstrated to map to multiple places in the genome) were filtered out [31]. The probes that were overlapping with rare SNPs (probes in transcription factor binding sites that showed extreme methylation pattern) were filtered out [32]. To minimize the unwanted variation within and between samples, we used the functional normalization method from the minfi.1.36.0 R package [33]. We ran a differential mean analysis using t-moderated statistics. Using the MEAL.1.20.3 R package pipeline, which, relies on the lmFit from limma R package (design model =  ~ Loading). CpGs after Bonferroni correction *P* < 6.243109e-08 (0.05/800883) were considered significant. Stratified analysis for each neo-cartilage construct was performed. These two datasets were then combined with a random effect meta-analysis using the metaVolcano R package The circos plot was produced using the Circlize 0.4.3 R package [34].

### Statistical analysis

For all data analysis except methylome data, we have used a generalized linear model including the factor hyper-physiological loading using R statistical software version 4.1.1.

### Supplementary Information

**Additional file 1**. Supplementary materials and methods.

**Additional file 2**. Supplementary tables.

## Data Availability

All data needed to evaluate the conclusions in the paper are present in the paper and/or the Supplementary Materials. Additional data related to this paper may be requested from the authors.
