# Supplementary material for: Hyper-physiologic mechanical cues, as an osteoarthritis disease-relevant environmental perturbation, cause a critical shift in set points of methylation at transcriptionally active CpG sites in neo-cartilage organoids
Source: Clin Epigenetics. 2024 May 10;16:64. doi: 10.1186/s13148-024-01676-0 (PMC11087253; doi:10.1186/s13148-024-01676-0)
Supplement: Supplementary file 1 — Additional file 1. Supplementary materials and methods. [file 13148_2024_1676_MOESM1_ESM.docx]

Supplementary Materials for

**Hyper-physiologic mechanical cues, as an osteoarthritis disease relevant environmental perturbation, cause a critical shift in set-points of methylation at transcriptionally active CpG sites in neo-cartilage organoids**

Niek G.C. Bloks et al.

Corresponding author: Ingrid Meulenbelt,

Email: [i.meulenbelt@lumc.nl](mailto:i.meulenbelt@lumc.nl)

**This PDF file includes:**

Figs. S1 to S3

**Other Supplementary Materials for this manuscript include the following:**

Tables S1-S3

**
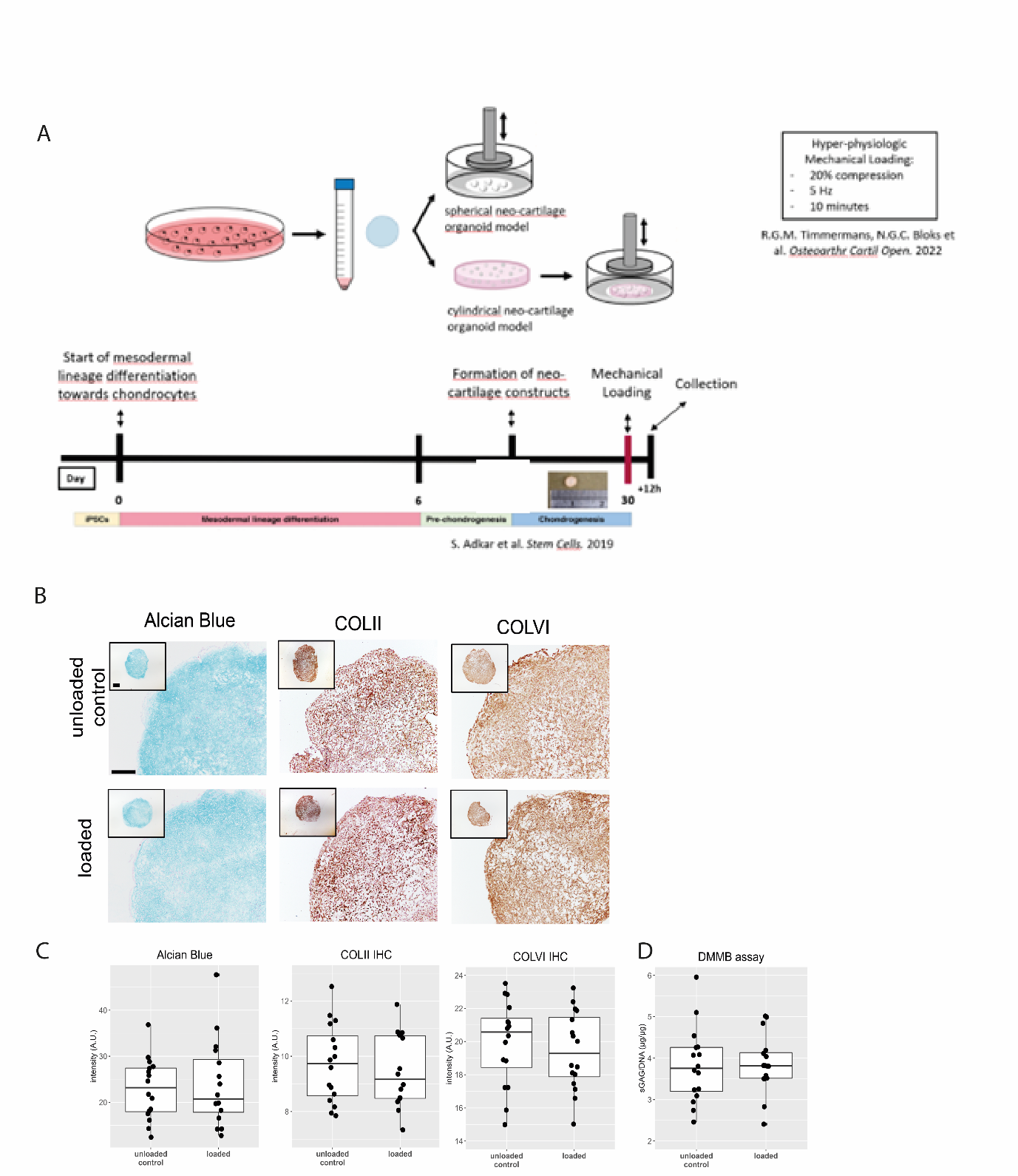
**

**Supplementary figure 1.Experimental set-up and effect of the hyper-physiological loading conditions on matrix deposition neo-cartilage organoids**. **(A)** Schematic representation experimental set-up adapted from Bloks et al. 2023, BioArxiv: hiPS cells were differentiated using an established differentiation protocol to produce neo-cartilage organoids. Two different organoid models were employed and jointly analyzed; 1. A spherical pellet model harnessing the original matrix produced by the hiPSCs. 2. A cylindrical organoid model in which the hiPSC-derived chondrocytes were embedded in an agarose construct, ideally suited for testing the effects of mechanical loading conditions. These constructs were both exposed to hyper-physiological loading conditions, after which the organoids were harvested for downstream analysis.

**(B)** Representative images of Alcian blue staining marking sulfated glucosaminoglycans (sGAGs) and immunohistological stainings of collagen II (COL II) and collagen VI (COLVI) in spherical neo-cartilage organoids. Scale bar 200µm. **(C)** Quantification of Alcian blue, COLII, and COLVI in unloaded controls and loaded spherical neo-cartilage organoids show no significant effect of hyper physiological loading (N=16). **(D)** Quantification of sGAG deposition in neo-cartilage organoids. sGAG deposition in these neo-cartilage organoids is not affected by mechanical loading. (N=16). Statistics are reported as beta ± standard error. The box plots represent 25th, 50th, and 75th percentiles, and whiskers extend to 1.5 times the interquartile range. Individual samples are depicted by black dots in each graph. P values were attained using a generalized linear model, with intensity (for immuno-stainings) and (sGAGs/DNA) as dependent variable and genotype as independent variable.

**
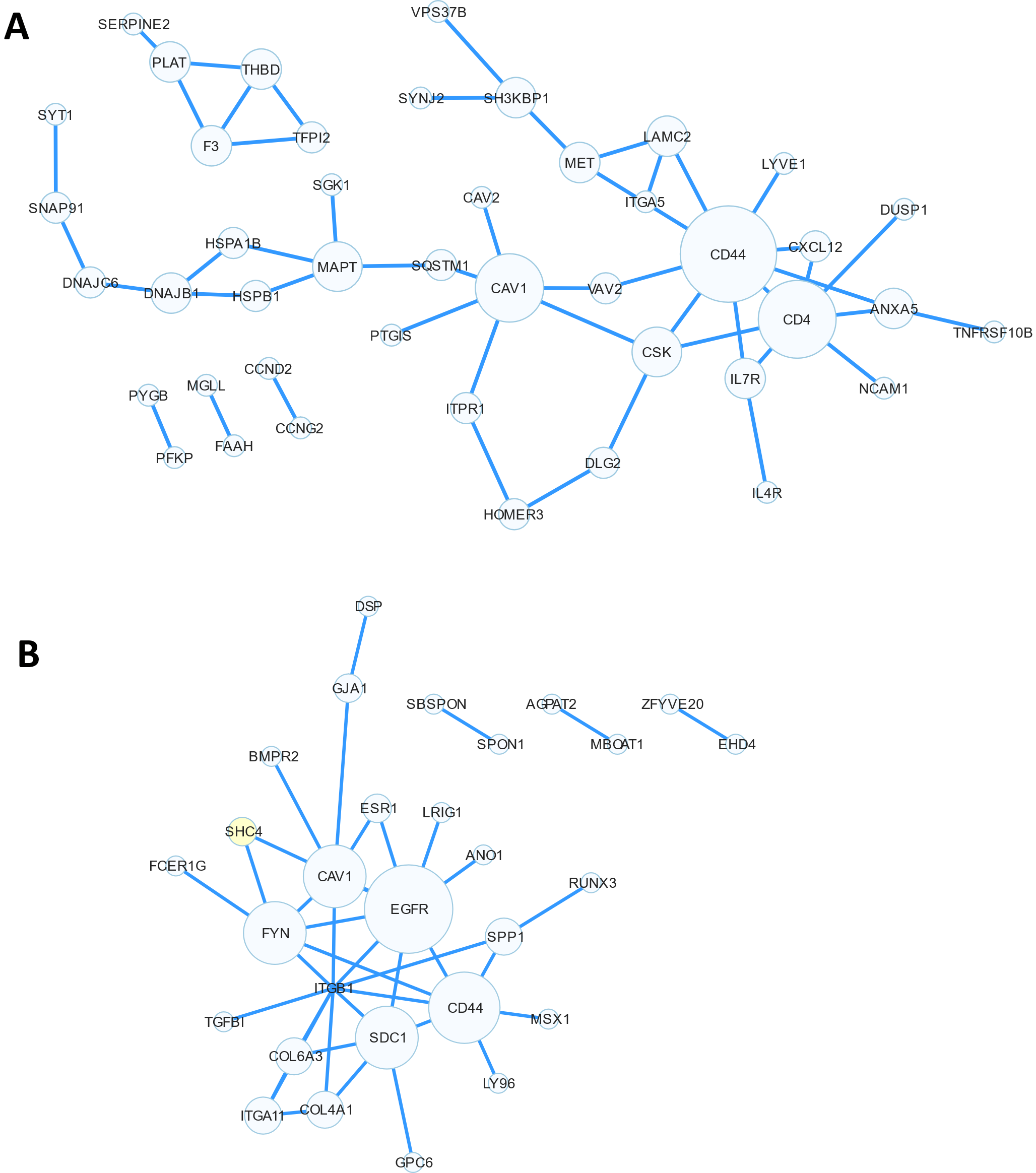
**

**Supplementary figure 2. Protein-Protein Interaction Networks** of (A) mechanically loading- or (B) OA-associated tCpGs as determined by STRING-DB.


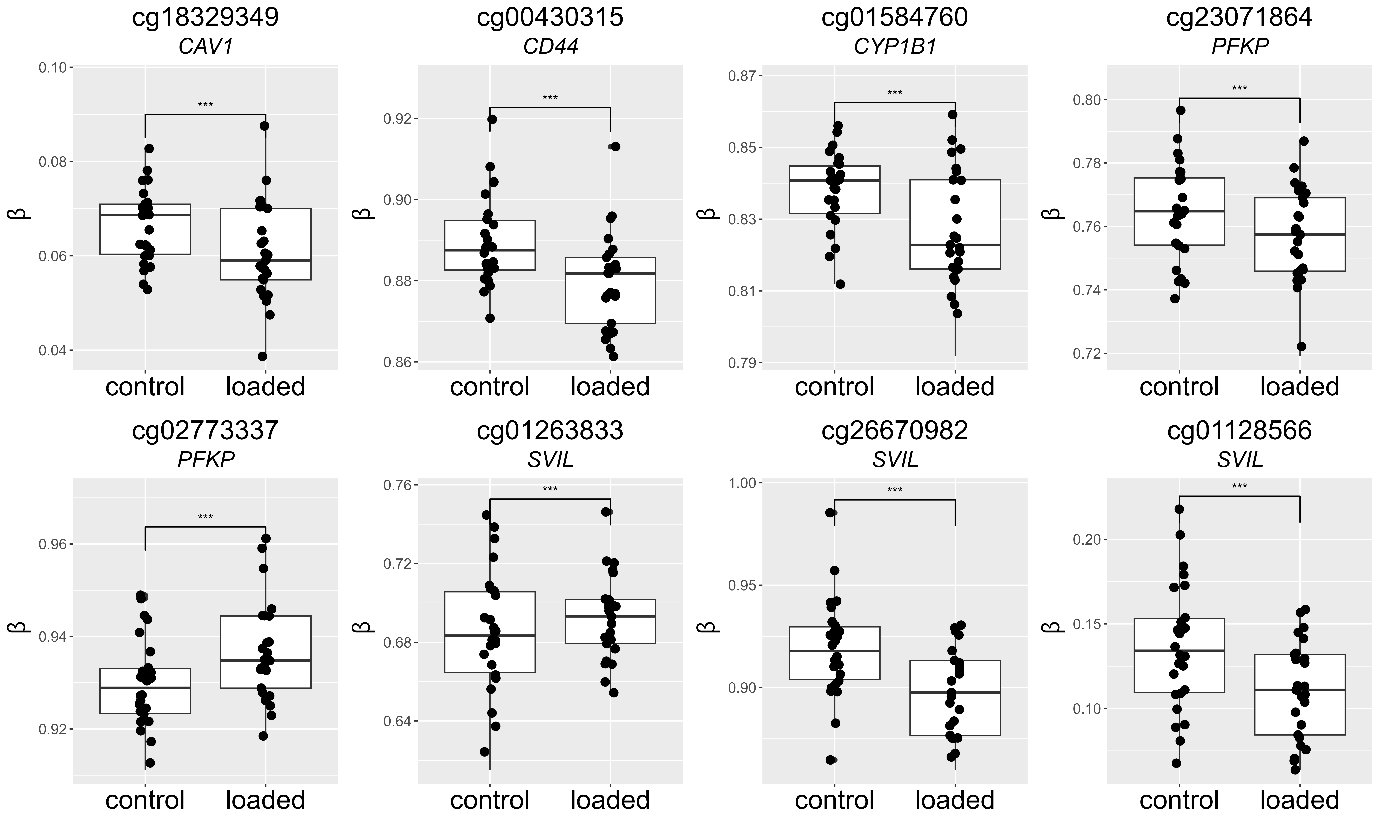


**Supplementary figure 3. Differential methylation of overlapping ML-tCpGs-genes with OA-tCpGs-genes.** Differential methylation of CpG sites mapped to the gene body. The box plots represent 25th, 50th, and 75th percentiles, and whiskers extend to 1.5 times the interquartile range. Individual samples are depicted by black dots in each graph. *FDR<0.05, ***FDR<0.001.
